# A Narrative Review on Managing Retinopathy of Prematurity: Insights Into Pathogenesis, Screening, and Treatment Strategies

**DOI:** 10.7759/cureus.56168

**Published:** 2024-03-14

**Authors:** Kratika Bishnoi, Roshan Prasad, Tanisha Upadhyay, Swapneel Mathurkar

**Affiliations:** 1 Medicine, Jawaharlal Nehru Medical College, Datta Meghe Institute of Higher Education and Research, Wardha, IND; 2 Ophthalmology, Jawaharlal Nehru Medical College, Datta Meghe Institute of Higher Education and Research, Wardha, IND

**Keywords:** neovascularization, anti-vascular, angiogenesis, prematurity, retinopathy

## Abstract

Retinopathy of prematurity (ROP) is a rare proliferative ocular condition that can happen in premature babies (born preterm <36 weeks) or who weigh <1.5 kg at birth (low birth weight babies). ROP is a major cause of childhood blindness. It is a premature disease since retina vascularization is completed only by 40 weeks of life. The survivability for preterm infants has increased owing to recent improvements in neonatal care during the past decade. As a result, the prevalence of ROP has risen concurrently. The abnormal development of blood vessels in the retina is the cause of this illness.

It occurs in two phases, phases 1 and 2. Most preterm infants weighing <1.5 kg need supplemental oxygen for respiratory support at birth. This leads to the initiation of phase 1 (vasoconstrictive phase). Phase 1 is characterized by loss of maternal-fetal connection and hyperoxia due to supplemental oxygen therapy. Oxygen's vasoconstrictive and obliterative action is primarily observed in developing retinal vessels. The inhibition of vascular endothelial growth factor follows from this. Phase 2 (vasoproliferative phase) shows the dilatation and tortuosity of the bigger existing vessels together with neovascularization and proliferation of new vessels into the vitreous when the baby is shifted from respiratory support to room air. Now, the retina gets hypoxic, where the retina becomes more metabolically active but is yet minimally vascularized, leading to VEGF-induced vasoproliferation, which might result in retinal detachment.

Patients with ROP face the danger of loss of vision. If correct and quick treatment is not provided, they might land into permanent blindness. Yet, ROP remains one of the most preventable causes of childhood blindness worldwide. Blindness caused by ROP can only be avoided if screening programs are readily available, pertinent, and appropriate. The initial stage in the therapy of ROP is the screening of premature neonates. Timely screening and management for ROP is important to avoid this irreversible loss of vision. The treatment is based on the severity of the disease. Management may include pharmacological interventions like intravitreal and anti-vascular endothelial growth factor and non-pharmacological interventions like laser surgery, vitrectomy, and scleral buckling.

We conducted a thorough literature search of studies on pathogenesis, risk factors, classification, and various treatment options for retinopathy of prematurity in infants, using a mixture of pertinent keywords. Only those studies published in peer-reviewed journals between 2010 and 2023 and written in English were included. Duplicate studies, unavailable in full-text for free, or studies unrelated to our subject matter were excluded. After thoroughly evaluating the selected studies, the results were synthesized and presented narratively. This article sheds light on the pathogenesis of ROP, particularly its relation to oxygen use, screening, and potential therapeutic management of ROP. Today advances in screening techniques have improved the outcomes for infants with ROP. Still, ongoing research is needed to optimize management strategies and reduce the burden of this condition.

## Introduction and background

Retinopathy of prematurity (ROP), earlier referred to as retrolental fibroplasia, was distinguished by a total retinal detachment (RD) behind the lens. It started appearing in preterm infants in the late 1940s. Using supplemental O_2_ in closed incubators increased the survivability of premature newborns but increased the risk of blindness; this was the root of the initial upsurge of ROP [1].

Low oxygenation levels have been linked to higher mortality. Yet, it stands unclear when and how much oxygen should be administered. Despite better oxygen supply management and advancements in other medical facilities worldwide, ROP is still a problem, even in the wealthiest countries. This epidemic of ROP is looming over middle-income countries because more premature infants now have access to neonatal intensive care units (NICU). This helped increase the survivability of premature infants in these nations, but without adequate oxygen management, these countries seem helpless. In some underdeveloped nations, 100% oxygenation therapy is still practiced, which is known to induce severe ROP even in older newborns [2].

Most cases of ROP were found in infants born at very low gestational age (<28 weeks at birth). With a preterm birth, an infant's retina is barely vascularized and has lower vascular endothelial growth factor (VEGF) and insulin growth factor 1 (IGF-1) levels than it should have in a case of full-term delivery. These immature retinas cannot grow normally due to the low availability of IGF-1 and VEGF. Thus, there is delayed vascularization, and the retina faces hypoxia. Hypoxia developing earlier than expected shows different impacts at different developmental stages [3]. By identifying postnatal factors that affect the risk for and the course of ROP, neonatologists, and ophthalmologists may be able to try to avoid the condition and reduce comorbidities with which it shares modifiable risk factors [4].

## Review

Methodology

**Figure 1:**
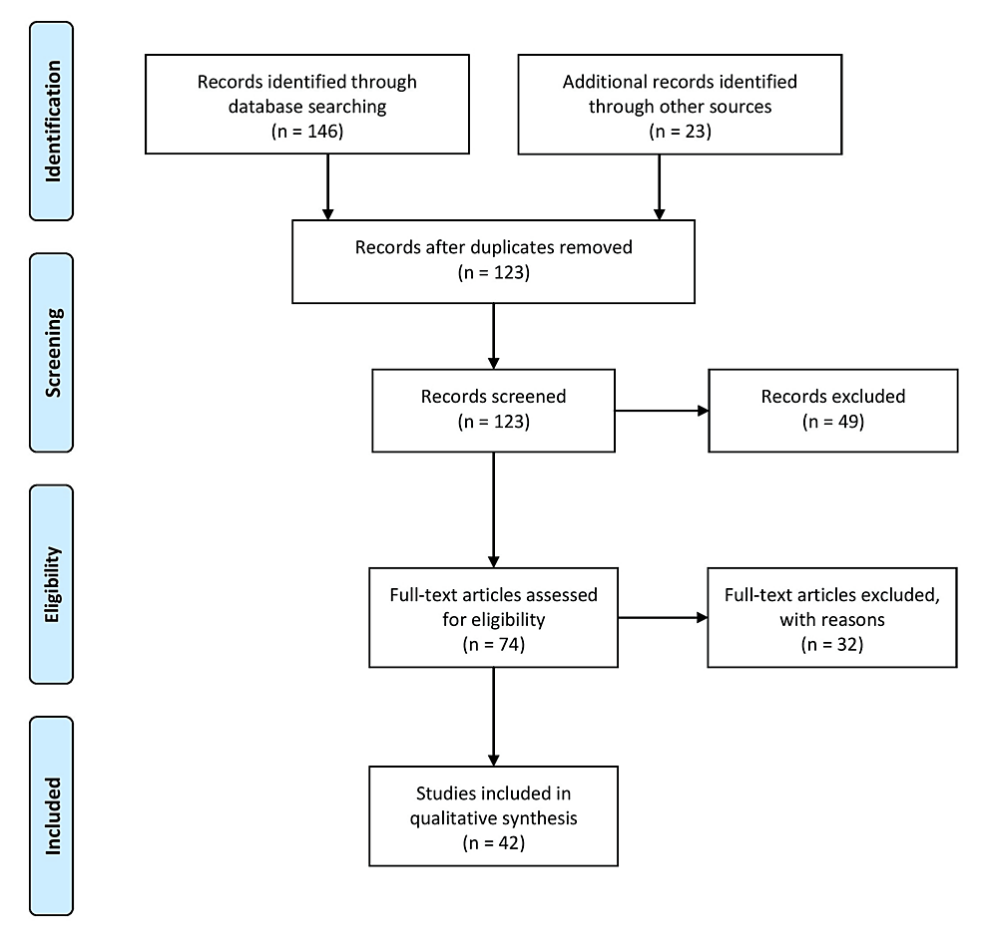
The selection process of articles used in this study. Adopted from the Preferred Reporting Items for Systematic Reviews and Meta-Analyses (PRISMA).

The methodology entails a thorough literature search of original articles, review articles, and reports published on the pathogenesis, risk factors, classification, and various treatment options for retinopathy of prematurity in infants. A mixture of pertinent keywords, such as “neovascularization,” “anti-vascular”, “angiogenesis”, “prematurity,” and “retinopathy” were employed. The literature search used Web of Science, Scopus, PubMed, and Google Scholar as the databases. Only those studies published in peer-reviewed journals and written in English were included. Articles published between 2010 and 2023 were included in our study to ensure the inclusion of recently published articles, which provide value for comprehensive research. Duplicate studies, unavailable in full text for free, or studies unrelated to our subject matter were excluded. Two separate reviewers scrutinized the titles and abstracts, and then the full texts of the chosen publications were evaluated. Conflicts were settled by consensus or, if necessary, by consulting a third reviewer. After thoroughly evaluating the selected studies, the results were synthesized and presented narratively. The method utilized to choose the papers for our investigation is depicted in Figure 1.

Pathogenesis

**Figure 2:**
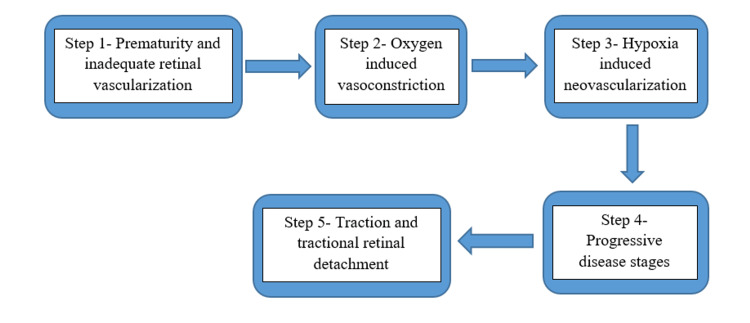
The pathogenesis of retinopathy of prematurity (ROP) can be understood in the following steps. The author self-created the figure using the information from [5].

The disruption of average neuronal and vascular growth in the retina, along with pathogenic compensatory mechanisms that lead to atypical vascularization of the retina, are associated with the pathophysiology of ROP. In the first trimester, hyaloid vasculature provides metabolic support for the retina and the inner portion of the eye. While the formation of vascular plexus starts at the optic nerve head and spreads centrifugally after eight months of gestation, reaching the nasal side, and after one month after delivery, reaching the temporal side, the transition from hyaloid to retinal vasculature begins by 16 weeks of gestation. In utero, a child is exposed to lower oxygen tension and proper vascular development, as premature-born infants are kept in the neonatal ICU (NICU) to support their respiratory function and exposed to excess oxygen, which is the leading cause of ROP. Excess oxygen supplement leads to vasoconstriction and vasoconstriction to hypoxia followed by the release of various growth factors VGEF, IGF-1 stimulates neovascularization. Abnormal blood vessel growth leads to tractional RD [5]. Figure 2 shows the pathogenesis of ROP.

ROP occurs in two phases, phases 1 and 2. Most preterm infants weighing <1.5 kg need supplemental oxygen for respiratory support at birth [6]. In phase 1, from birth to approximately 30 weeks postmenstrual age, retinal vascularization is restricted by hyperoxia (due to supplemental oxygen therapy), a lack of nutrition, and growth hormones from the mother-child interface. Blood vessel growth decreases as the retina ages and metabolic demands increase, leading to hypoxia [7-9]. When the infant is shifted from respiratory support to room air, the hypoxic retina is brought on by phase 2 oxygen-regulated molecules such as VEGF and erythropoietin (EPO) that work to stimulate neovascularization of the retina. After preterm birth, IGF-1 levels gradually rise from extremely low to sufficient levels to activate VEGF pathways. Preventing phase 1 by increasing IGF-1 to levels reported in pregnancy and lowering O_2_ to avoid inhibiting VEGF can restore retinopathy. In the alternative, phase 2 following neovascularization may be accomplished by suppressing the VEGF level using laser therapy or an antibody [10,11]. Finding the ideal postnatal environment for these underdeveloped babies may be possible by becoming aware of these phases and their reasons [12].

*Phase 1 (Vasoconstrictive Phase* *)*

The vascular growth stops, mainly caused by hyperoxia. In contrast to the prenatal environment, where mean O is below 50mm mercury (Hg) throughout the second part of pregnancy, even room air might result in hyperoxia [7]. Furthermore, providing additional oxygen to premature infants experiencing respiratory distress might result in unusually high oxygen saturation. In particular, EPO and VEGF are oxygen-regulated angiogenic growth factors suppressed by hyperoxia. This results in the stop of retinal vessel formation and the depletion of certain existing retinal vessels. In better-developed babies, exposure to high oxygen concentrations lowers preexisting vasculature that is not seen with a controlled oxygen supply, which largely prevents vessel creation, claim a few studies [8].

Some variables like IGF-1, typically present in utero at optimal quantities and absent in human newborns born before the end of the third trimester of pregnancy, may also be responsible for stopping vascular expansion. IGF-1 is essential for numerous tissues, involving the brain and blood arteries, to grow and develop normally. The emergence of ROP is believed to be influenced by the mother’s deficiency in long-chain polyunsaturated fatty acids [9].

Phase 2 (Vasoproliferative Phase)

Phase 2 starts while the retina, rendered hypoxic due to suppression of vessel formation in phase 1, becomes more metabolically active but less vascularized. Neovascularization in the retina is the characteristic feature in phase 2, primarily due to an increase in VEGF and EPO brought on by hypoxia. RD and fibrous scar formation are caused in newly developed arteries due to their poor perfusion and leaky construction [10]. ROP typically develops independently in most newborns, though neurological impairments (loss of photoreceptor function) might persist even in mild cases. Phases 1 and 2 appear to switch over depending more upon the infant’s postmenstrual age than its postnatal age. Irrespective of the gestational period at delivery, the disease in a group of babies with birth weights less than 1,251 grams began to manifest about 30 weeks postmenstrual age and peaked at 36-38-week postmenstrual period. This significant study reveals that the postmenstrual age is more strongly linked with the beginning of ROP than the postnatal age, suggesting a link between the scheduled chronology of development and disease pathogenesis [11].

In highly preterm cases, this relationship could not be readily noticeable. According to a study of newborns with gestational ages between 24 and 26 weeks and seven days old at birth, the development of retinal vascular episodes was significantly more correlated with a postnatal period (mean range eight to nine weeks) rather than postmenstrual age, suggesting that extreme prematurity increases the risk of developing ROP. Vascular complications arose more quickly in more immature babies. Stages of ROP may occur at different rates depending on the degree of exposure to high oxygen concentrations. A recent study discovered that several mature preterm babies who were exposed to 100% oxygen after delivery {gestational age at birth: 31.7 week (range: 28-35 week) lost their retinal arteries (stage 1) and advanced to considerable zone-1 neovascularization (stage 2). Preterm birth-related variables may occasionally impact intrauterine retinal neurovascular development. The fetal retina may be predisposed to severe ROP by antenatal variables like placental infection and inflammation, and this sensitization effect may be the prophase of the disease [12].

Compensatory Mechanisms Leading to Abnormal Vascularization

Phase 1 shows hyperoxia-induced damage leading to retinal microvascular degeneration in the PR. Phase 2 of the disease begins when the hypoperfused tissue cannot fulfill the growing metabolic demands of the developing retina. Growth factors released during hypoxia cause aberrant neovascularization, which causes vessel growth to be misdirected from the retina into the vitreous. Fibrovascular traction can result in partial or complete RD, seriously impairing vision [13].​​​​​​

Increased retinal eNOS expression in retinal hypoxia leads to increased nitrogen oxide (NO) production, which in turn causes vasodilation and angiogenesis. Vasodilation brought on by NO acts as a compensatory strategy to lessen vascular obliteration during the early phases of ROP. Reactive oxygen species (ROS) are produced due to the improvement in ocular blood flow that follows. Moreover, NO plays a crucial role in retinal endothelial cells, raising vascular permeability and pathological neovascularization even though it is necessary for quickly starting angiogenesis. It has been reported that vascular growth factors in ROP work compensatively. Here, VEGF expression inhibition results in the up-regulation of other angiogenic factors such as basic fibroblast growth factor (bFGF) and angiopoietin (Ang-1), whereas endothelial cells under hypoxic conditions exhibit the opposite pattern of VEGF up-regulation. This mechanism may help to explain why a combination approach may be more successful in preventing the recurrence of neovascularization in ROP than the selective suppression of a single growth factor. It has been reported that vascular growth factors in ROP have a compensating mechanism, which helps to explain why blocking a single growth factor is ineffective in stopping the recurrence of neovascularization [6,14].

Emergency of ROP

Classification of ROP

ROP is typically classified into stages based on the severity of the disease. The system commonly used is the international classification of ROP (ICROP). Table 1 describes the classification of ROP into five main stages. The classification helps us to determine the appropriate management and treatment for infants with ROP. Premature infants must receive regular eye examinations for successful diagnosis and early treatment of ROP [15].

**Table 1:** Classification of retinopathy of prematurity. The author recreated the table using information from [15].

Stage	Severity	Changes seen on the retina
1	Mild	A line demarcating normal and abnormal blood vessel growth appears.
2	Moderate	Growth of abnormal blood vessels into retina.
3	Severe	A further progression of abnormal blood vessel growth forms a tissue mass. This stage is subdivided into three zones (zones I, II, and III) based on proximity to the retina's centre.
4	Advanced	Abnormal blood vessel growth leads to partial retinal detachment (RD).
5	Total RD	Retina is completely detached, causing significant vision loss or blindness.

Risk Factors

Oxygen: Throughout ROP, oxygen has an exceedingly complicated role. While rapid drops in oxygen levels set off compensating processes that encourage the creation of aberrant blood vessels, elevated initial levels of oxygen can lead to oxidative stress and compromise normal retinal blood vessel development. Oxygen levels must be carefully managed to reduce the chance of ROP developing in premature infants [16].

Gestational age and birthweight: Prominently associated risks for retinopathy of prematurity include low birth weight (LBW) for gestational age and born for LGA. These two factors affect how immature the retina’s neuronal and vascular development is at birth, affecting how vulnerable the retina is to injury [16].

IGF-1 level: Early-postnatal low serum IGF-1 concentrations are associated with subsequent ROP and prematurity-related complications in preterm infants. Low IGF-1 serum levels in preterm newborns are linked to slower head circumference growth and are directly correlated with the extent of retinopathy of prematurity [17].

Postnatal weight gain: IGF-1 substantially correlates with preterm children’s postnatal weight increase. Hyperglycemia, insulin, and increased glucose levels in newborns also raise the chance of developing prematurity-related retinopathy. Insulin usage and hyperglycemia were linked to a rise in both severe and less severe ROP [17].

Genetic factors: Additionally, ROP danger may be influenced by inherited characteristics. Infants who are white or black are more likely to contract the disease, as are boys than girls. Another risk factor for ROP is infections, which more commonly attack newborns, especially fungal ones. Blood transfusions also pose a potential threat to the development of ROP [17].

Screening of ROP

The ROP screening recommendations serve two purposes. First, to recognize babies at risk for ROP and precisely examine their retinal development after birth. Second, to recognize babies who have severe illness and need treatment. All infants at risk for ROP should be examined as soon as possible because ROP can lead to permanent vision loss if not diagnosed in time. Detecting the change from stage I to II, or reinstatement of retinal vascular development, is the main objective of ophthalmologic screening for ROP [18].

Screening is typically recommended starting at the sixth postnatal week or 31 weeks postmenstrual age. An ophthalmologist enlarges the infant's pupils with eye drops while screening and checking the retina with a retinal camera or a specialized ophthalmoscope. The objective is to find aberrant blood vessel development or other ROP symptoms. Inspections are conducted every one to two weeks during the screening process until the ROP entirely declines or the developing retinal blood vessels develop fully, usually between 40 and 50-week postmenstrual age [19].

The severity and development of the ROP detected during the initial test determine the number and timing of subsequent examinations. If ROP is found, the ophthalmologist evaluates the extent of the problem and chooses the best course of treatment, which may include close observation, laser therapy, or surgery. The goal of treatment is to halt ROP’s progress and lower the probability of vision loss or blindness. For premature newborns with a previous diagnosis of ROP, routine follow-up exams beyond infancy are frequently advised to evaluate their eye health and identify any potential long-term issues. To achieve the best visual outcomes for premature newborns, prompt and thorough screening is essential for diagnosing and controlling ROP [20].

In a study by Popoola et al., in Nigeria, 723 infants were examined; out of them, 127 (17.6%) developed any ROP, and 29 (22.8%) out of 127 developed type 1 ROP [21]. In another study by Hakeem et al., out of the 172 newborns evaluated, 33 infants (19.2%) developed ROP in one or both eyes; the breakdown of cases was 18 (54.5%) for stage 1, nine (27.3%) for stage 2, and six (18.2%) for stage 3. While no infant was diagnosed with stage 4 or stage 5 ROP [22]. A study of 62 preterm newborns was conducted by Hegde et al., six newborns (9.68%) out of the 62 developed ROP; of these, four babies (66.67%) had stage 1 ROP, one baby (16.67%) had stage 2 ROP, and one baby (16.67%) had stage 3 ROP. At the same time, no infant was diagnosed with stage 4 or stage 5 ROP [23].

In a study by Athikarisamy et al., they concluded that the primary benefit of routine screening is a significant increase in “lead time,” which is the amount of time that passes between the time at which alterations become detectable and the period at which the disease would have manifested symptomatically if screening had not identified it. This is especially true for ROP since symptoms do show up in newborns or parents until the retina separates [24]. ROP screening coverage and quality improved after cooperative efforts on a national and international level. Systems for data monitoring and equipment for ROP treatment and neonatal care are desperately needed to scale up and enhance services. Sustained advocacy is also necessary [21].

Treatment

Pharmacologic Treatment: Anti-VEGF Therapy

Bevacizumab: The preliminary anti-VEGF medication being looked into for ROP is bevacizumab, a recombinant humanized antibody for colon cancer that has received FDA approval. By this, bevacizumab eliminates the angiogenic threat of ROP (BEAT-ROP) research; bevacizumab is more successful than laser therapy in treating children with zone I, stage 3 ROP+ disease. Compared to infants receiving laser therapy, those given intravitreal bevacizumab experienced fewer ROP instances of recurrence and worse ocular findings, such as macular dragging. In a subsequent clinical trial of BEAT-ROP, the patients from the group who received bevacizumab, experienced fewer instances of high myopia in comparison to those who received laser therapy [25,26].

Ranibizumab

Ranibizumab, a monoclonal immunoglobulin recombinant antibody, and yet another anti-VEGF inhibitor, recently earned its first European approval for treating ROP. The FDA has approved ranibizumab as a therapy for wet age-related diabetic retinopathy and macular degeneration. Bevacizumab’s binding for VEGF is less than ranibizumab’s, and it also has fewer systemic adverse effects and a shorter half-life. Ranibizumab therapy causes ROP to return more frequently than bevacizumab does. Additionally, in a randomized controlled trial, the potency of laser therapy was compared to that of high-dose (0.2 mg) and low-dose (0.1 mg) ranibizumab [27]. Individuals in the high dose ranibizumab group reported fewer ocular adverse effects. They had a greater rate of treatment effectiveness compared to those in a laser therapy group, suggestive of the superiority of high-dose ranibizumab therapy to anti-VEGFs provides numerous benefits over lasers, such as more rapid and simple administration, lesser destruction of structures, less refractive error, and potential treatment in unique circumstances such as cloudy media, non-dilating pupil, corneal opacification, and posterior pole ischemia [28].

Inconvenient variables regarding the precise dose needed, follow-up frequency and duration, recurring detection and management, continuing peripheral avascularity, prolonged impacts on visual acuity, and systemic side effects, notably those associated with neurodevelopmental delay, are among the potential drawbacks. Legal and medical issues are also quite important in countries like India. Even though the Indian government banned and then reintroduced anti-VEGF medications used to treat adult retinal issues, there is still no appropriate punishment for using these medications on newborns, which could expose the treating physician to legal risk. Rural patients may be unable to follow up consistently following treatment, which puts them at a significantly higher threat for late, undiscovered, and untreated recurrences [29].

The fact that VEGF concentrations are reduced in type 1 ROP patients for two to three months after intravitreal anti-VEGF injections leads to systemic side effects of anti-VEGF therapy. This is likely because the drug leaked into the systemic circulation. Compared to preterm newborns treated with laser, those treated with bevacizumab had a greater risk of developing acute neurodevelopmental impairments, according to retrospective observational research. They did not discover that infants who got only bevacizumab, as opposed to those who underwent laser photocoagulation, had poor neurodevelopmental results [28,29].

Laser Surgery

Laser surgery is the most common procedure for ROP treatment, where the peripheral retina (PR) is scarred by tiny laser beams (also known as laser therapy or photocoagulation). The laser procedures are carried out in a hospital’s newborn unit, operating room, or another location during which a doctor or anesthetic may continue to monitor the child. The 532 nm green laser has largely supplanted the diode laser in many centers. The formation of scar tissue formation on the periphery of the retina is the goal achieved through cryotherapy and laser surgery. The PR scar will no longer promote the development of aberrant vessels [30]. Following the surgery, normal retinal vessels will develop during which they were absent previously. Despite the injured area of PR no longer functioning, the central retina will continue to operate correctly, and vision can be maintained. Typically referred to as the ROP procedure, laser surgery uses a light beam to burn the scarred tissue of PR. The ophthalmologist uses the laser to direct light beams at scorching and eventually scarring the PR area where these erratic blood vessels are still not entirely developed. Each eye undergoes laser surgery for an average of 30-45 minutes. The child’s eyes and eyelids will probably appear red after the procedure. One can also notice slight puffiness near the eyelids [31]. That could linger for a few days to a few weeks and is normal. The infant will be prescribed eye drops for a week, and the eye won’t be covered. Numerous benefits of the latter procedure were noted, including reduced pain, less tissue penetration, ease of usage, lower cost, and enhanced portability. Diabetes retinopathy is another condition that the green laser can cure, and in low- and middle-income nations, this has a cost-utility advantage [32].

Cryotherapy

Extremely cold temperatures scar the retina’s periphery. Cryotherapy, often known as cryosurgery, was the standard procedure for ROP surgery for a long time, but laser therapy has supplemented it. These techniques are utilized for more severe ROP situations where RD occurs. The scleral buckling procedure entails wrapping an elastic band, frequently made of silicone, over the eye’s rim. The band is wrapped around the eye’s white or sclera, causing it to “buckle” or push inward. As a result, the ruptured retina is pushed up against the eye’s outer border and continues to do so [33].To prevent the growth of aberrant blood vessels, cryotherapy freezes and scars the PR. During a cryotherapy operation, the sclera is touched with a metal probe exposed to liquid nitrogen, a very cold gas. The PR is then abruptly exposed to the cold, which freezes and damages the area [34]. A cryotherapy session lasts 30-45 minutes. The infant’s eye will likely be red, and a certain swelling may surround the eyelid. The infant will be prescribed eye drops for a week, and the eye won’t be covered.

Scleral Buckling

Scleral buckling is only used in ROP situations where the retina has already started to separate. It is performed on patients with stage 4 ROP who only have PRl traction. It entails placing 240 bands at the height of the tractional RD (TRD) by making scleral tunnels in each quadrant. The concept is to wrap a band over the side of the retina instead of the lens. The retina is forced back in its place by this band [35].

A retinal surgeon performs this surgery. The surgeon initially removes the aberrant blood vessels using laser surgery or cryotherapy. The detached retina is then covered by a band, frequently composed of silicone, which has been wrapped over the sclera. The surgeon may make a minor incision to remove excess fluid if there is too much of it under the detached retina. The band is then sewn into position [36]. The newborn’s eyes and the region surrounding the eyelids will be red and swollen during the scleral buckling process, lasting one to two hours. A patch will be applied over the eye until it has healed. The buckle should be checked on patients who have had scleral buckling every half-yearly or more frequently if a patient has any complaints. It is removed if the buckle is overly tight or poses an issue. It will eventually be eliminated to make room for the developing eye (months or years later) [37,38].

Vitrectomy

A difficult procedure in which a saline solution substitutes the vitreous solution. Scar tissue can be removed, and the pull on the retina is lessened by removing the vitreous. This prevents the retina from separating. Lens-sparing vitrectomy (LSV) is the most frequent procedure for stage 4 ROP. After vitrectomy, the eye's vitreous humor is exchanged by a saline solution that functions almos0t as vitreous. A retinal surgeon can ease the pressure on the retina by removing internal scar tissue using a vitrectomy [39]. Utilizing a scalpel, the surgeon makes multiple tiny incisions in the sclera while utilizing a microscope on the eyeball to observe the insides of the eye. The incisions are used to introduce a small cutting device and an infusion line to maintain the intraocular pressure to preserve the structure of the eye. Along with vitreous, any blood, scarred tissue, or other debris inside the eye is removed. The saline solution is replaced with vitreous via the infusion line. Then, bio-absorbable sutures are used to close all incisions. The procedure of vitrectomy can take an entire day. The patient will experience some reddenedness and swelling near the eyelids and in that region around the eyes. A patch will be applied over the eye until it has healed [40]. A newborn with a vitrectomy will take several weeks to recover and require regular eye checkups.

Complications

Although unmanaged ROP can cause blindness due to RD, individuals who have received treatment are at risk for several problems. These dangers may endure for the rest of the patient’s life. Myopia, one of the most frequent consequences of ROP, can worsen throughout childhood. Additionally, the macula might drag, resulting in pseudostrabismus and poor vision. After receiving early treatment, patients of ROP have more risk of developing glaucoma, cataracts, amblyopia, strabismus, and RD [34]. These kids need to be continuously watched throughout childhood to catch any late difficulties [41].

Strabismus (12.8%), nystagmus (3.3%), epiphora (0.6%), corneal opacity (0.6%), cataract (0.3%), lid fissure asymmetry (2.4%), and corneal diameter asymmetry (2.0%) are among the side effects of cryotherapy. Compared to eyes receiving laser treatment, eyes treated with cryotherapy have a larger rate of unsatisfactory structural and functional outcomes [42]. When treating severe ROP, laser therapy is more likely to produce poor structural results and myopia than anti-VEGF treatment. According to clinical trial statistics, 1.4%-3.6% of anti-VEGF treated eyes and 9.1%-9.5% of laser-treated eyes had poor outcomes. According to additional randomized trial data, zone I eyes treated with bevacizumab had a risk of 3.8 and 51.4% for very high myopia (≥-8.00D), respectively. However, unlike lasers, anti-VEGF is more likely to require retreatment due to the possibility of late recurrence. General anesthesia is frequently required for laser procedures, which carries its hazards, including worse short-term respiratory results [41].

The two commonly used methods now have significantly distinct treatment consequences. Anti-VEGF treatment is preferred in more severe instances due to the danger of poor structural results and myopia. The risk of recurrence and the requirement for further treatments may benefit laser in less severe cases of ROP. Studies that have been conducted using anti-VEGF have shown neurodevelopmental problems. However, more information is required to determine the relative risks of neurodevelopmental problems [41].

## Conclusions

ROP is a complicated ophthalmic condition that can create serious problems for premature babies. ROP can result in the growth of aberrant blood vessels, RD, and irreversible vision loss or blindness if it is not treated. ROP treatment may also lead to other issues, such as strabismus, amblyopia, myopia, astigmatism, cataracts, and glaucoma. To reduce the effects of these issues and enhance visual outcomes after treatment, it is essential for premature infants and LBW infants to undergo routine eye exams and the proper therapy. To create prevention and burden-reduction measures, further knowledge of the epidemiology and etiologies of ROP is required. Improving oxygen techniques is one of the main preventative measures. ROP screening is essential for identifying at-risk newborns and initiating interventions to protect preterm infants' visual health. The issues posed by ROP must be managed and addressed with timely intervention and continued care.
